# Effect of basin water depth on the performance of vertical discs’ solar still—experimental investigation

**DOI:** 10.1007/s11356-022-22220-8

**Published:** 2022-07-27

**Authors:** Mohamed Ragab Diab, Fawzy Shaban Abou-Taleb, Fadl Abdelmonem Essa

**Affiliations:** Mechanical Engineering Department, Faculty of Engineering, Kafrelsheikh University, Kafrelsheikh, 33516 Egypt

**Keywords:** Vertical disc distiller, Rotating parts, Desalination, Saltwater depth, Solar still modifications

## Abstract

The ability to get clean water is the most urgent birthright for human beings. The scarcity of safe drinking water is a major challenge in both developed and developing countries. Due to overpopulation, industrial revolution advancements, and agricultural evolution, this challenge has become crucially influential. Several studies on solar desalination are being conducted to create novel models that will improve the efficiency and production of these units. Because of their higher evaporation, condensing, exposure, and output rates than traditional stills, vertical distillers have lately piqued the interest of numerous academics. In this study, the scholars investigated the impact of varying water depth at the best rotating speed of discs from their earlier work (1.5 rpm) on the thermal productivity of vertical distillers. Numerous water depths (5, 8, 11, and 14 cm) were studied at 1.5 rpm to specify the best depth. The results indicated that utilizing moving discs enhanced the distillers' productivity. Besides, the peak distiller performance was obtained at 1.5 rpm and 5 cm. Furthermore, the yield of the modified single-stage vertical distiller (MSSVD) and modified double-stage vertical distiller (MDSVD) was increased by 350 and 617.4%, respectively, over the conventional tilted distiller (CTD) productivity of 2.3 L/m^2^ day. MSSVD and MDSVD had the highest efficacy rates of 48.4 and 77.2%. Lastly, for CTD, CVD, MSSVD, and MDSVD, the pure water cost was 0.025, 0.0477, 0.0180, and 0.0193 $/L, respectively.

## Introduction

One of the most well-known challenges of the twenty-first century is freshwater resources shortage, which has several negative consequences for humanity (Essa 2022; Saleh et al. 2022). The globalization of the water crisis has had a greater influence on the lives of those living in rural and isolated places, as well as on the lives of the poor who cannot afford to use high-tech to obtain water (Diab et al. 2022; Omara et al. 2021; Tiwari and Sahota 2017). Basic medical services were unavailable in some areas in developing and impoverished countries. Several rural people are still unsure about the consequences of consuming untreated water (Felemban et al. 2022; Kalogirou 2005; Pugsley et al. 2016).

Solar distillers are simple devices and do not require advanced expertise (Elango et al. 2015; Panchal et al. 2020), although they have low productivity (Bouchekima 2003; Elashmawy 2020). This inspired researchers to figure out new ways to boost distiller productivity, focusing their attention on amending previous designs and introducing innovative models to enhance and strengthen distiller production and thermal performance through research on multiple parameters such as expanding evaporation surface area, heat transfer process, and water level minimization (Nafey et al. 2000; Tiwari and Tiwari 2006). As a result, the literature on solar stills has a variety of designs and adjustments, all of which are targeted toward improving the solar still’s performance. The modifications are like rotating wick distiller (Abdullah et al. 2019a), solar stepped still (Abujazar et al. 2018a, 2018b), drum distiller (Abdullah et al. 2019b), disc solar still (Essa et al. 2020), hemispherical solar still (Attia et al. 2022), dish distiller (Saleh et al. 2022), solar still incorporated of the condensation unit (Kabeel et al. 2014b), pyramid solar still (Farouk et al. 2022; Kabeel 2009), solar still with reflectors (Omara et al. 2016), tubular solar distiller (Essa et al. 2022), and distiller using the fins (Omara et al. 2011), half-barrel (Younes et al. 2021) and corrugated surfaces (Omara et al. 2015), and nano-materials (Abdelgaied et al. 2022; Bait and Si–Ameur M 2018; Kabeel et al. 2014a).

Solar distillers integrated with moving parts such as rotary fans, drums, discs, or shafts outperform conventional distillers (Diab et al. 2021; Kabeel and El-Agouz 2011; Singh et al. 2021). The rotational element reduces surface tension and blends the free evaporation approach into the forced evaporation process (Katekar and Deshmukh 2020; Sharshir et al. 2016). These effects can increase the rate of evaporation. In a study, Omara et al. (2017) investigated a fan with differing water depths in a conventional distiller. They declared that for low rpm, the water layer should be minimum, and vice versa. They raised the yield by 17%. Kabeel et al. (2012) incorporated a vertical rotating fan into the basin to improve distiller output. The main idea of installing a rotary fan was to eliminate basin surface tension and convert natural to forced heat transfer. At a water depth of 3 cm, the optimum fan speed was 45 rpm, which caused a 25% improvement in output. Further, the idea that Abdullah et al. (2019b) and Malaeb et al. (2016) was to incorporate a movable drum into the basin, added 350% and 250% to an increase in the distillate. Essa et al. (2020) have recently presented a revolutionary distillation technique, combining the rotating discs with a standard distiller. The analysis found that the distilled water production was higher than that of the conventional distiller. Moreover, the distilled water output of the wick corrugated disc still was 124% higher than the standard distiller. For flat and corrugated discs with wick, the total thermal efficacy at 0.05 rpm was 50% and 54.5%, respectively.

Haddad et al. (2017) studied the vertical rotating cloth belt inside the solar still under the light of other movement arrangements in the distiller. In the winter, productivity increased by 51.1%. In addition, Gad et al. (2011) benefited from a moving horizontal wick belt inside the still basin to achieve maximum potable water output. Abdullah et al. (2019a) have analyzed the vertical and horizontal rotating impact of a cloth belt in varying periods of OFF times inside the distiller. They also noticed that the optimum productivity is 30 min OFF. The drinking water distillation was improved to 315% with the use of nanoparticles. Furthermore, a rotating shaft was mounted inside the distiller in order to maximize the distillate yield (Abdel-Rehim and Lasheen 2005). The analysis showed an improvement of 2.5% in May, 5% in June, and 5.5% in July in thermal efficacy. As well, Kumar et al. (2016) studied the effect of using a water agitator in the still basin which led to enhancing the distiller’s productivity by 39.49% compared to the traditional still.

Taking into consideration a different arrangement within the outline of the glass cover still associated with it. Mohammed and Hashim (2013) covered the vertical still reservoir with a black cloth wet to distillate water and incorporated it with the external reflector. Vertical solar still output (VSS) without any improvement was 17.6% and improved to 38.2% via an exterior reflector (VSSR).

In comparison to previous concise literature and recent work (Diab et al. 2022, 2021; Essa et al. 2021), incorporating rotating discs in a vertical distiller significantly increased the distillate yield of the still. In this study, the scholars aimed to investigate the impact of varying water levels on still performance to answer some critical questions: “would the distillate yield of the modified distillers be enhanced if the water depth was increased? Does expanding the wet area of the discs boost the evaporation rate and counteract the drawbacks of raising the basin water depth?” The basic principles of this analysis are to reduce saltwater film on rotating discs, capture radiation from the sun as much as possible, and break the surface tension. The key purpose of this research is to enlarge the surface area of the water influenced by sunlight rather than traditional solar stills. Consequently, the novelty of this research can be highlighted as follows:

1. A thin rotary flat disc inside the vertical distiller has been tested, where the low disc side is partially immersed in the saltwater reservoir, enhancing the surface of evaporation and the solar radiation exposure to water in the basin.
2. In addition, it was important to change the layout of the glass cover to accommodate rotating discs. The modified glass cover also helped to absorb extra sunlight and tracked to the sun even though the still was motionless.
3. Finally, the performance of vertical distillers incorporated with rotating discs was investigated at various water depths (5, 8, 11, and 14 cm) at the best rotational speed (1.5 rpm (Essa et al. 2021)).

## Materials and methods

### Experimental set-up

**Fig. 1:**
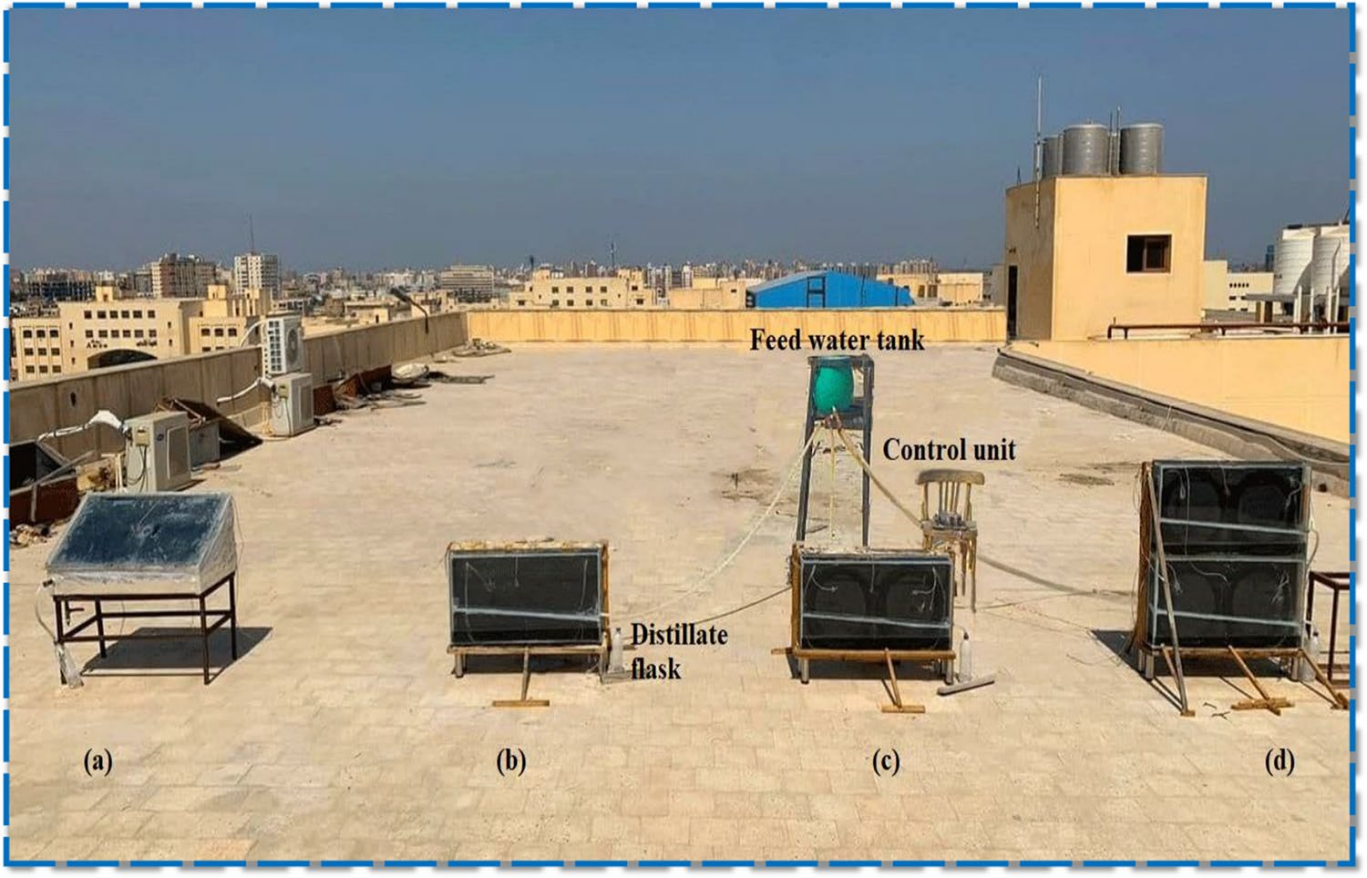
Photographic view of the experimental test-rig. **a** CTD. **b** CVD. **c** MSSVD. **d** MDSVD

The photograph of the experimental test-rig, as illustrated in Fig. 1, showed four distillers [conventional tilted distiller (CTD), conventional vertical distiller (CVD), modified single-stage vertical distiller (MSSVD), and modified double-stage vertical distiller (MDSVD)] as well as a feedwater tank that fed all the solar stills with water. The performance of MSSVD and MDSVD was evaluated and compared to that of CTD and CVD.

**Fig. 2:**
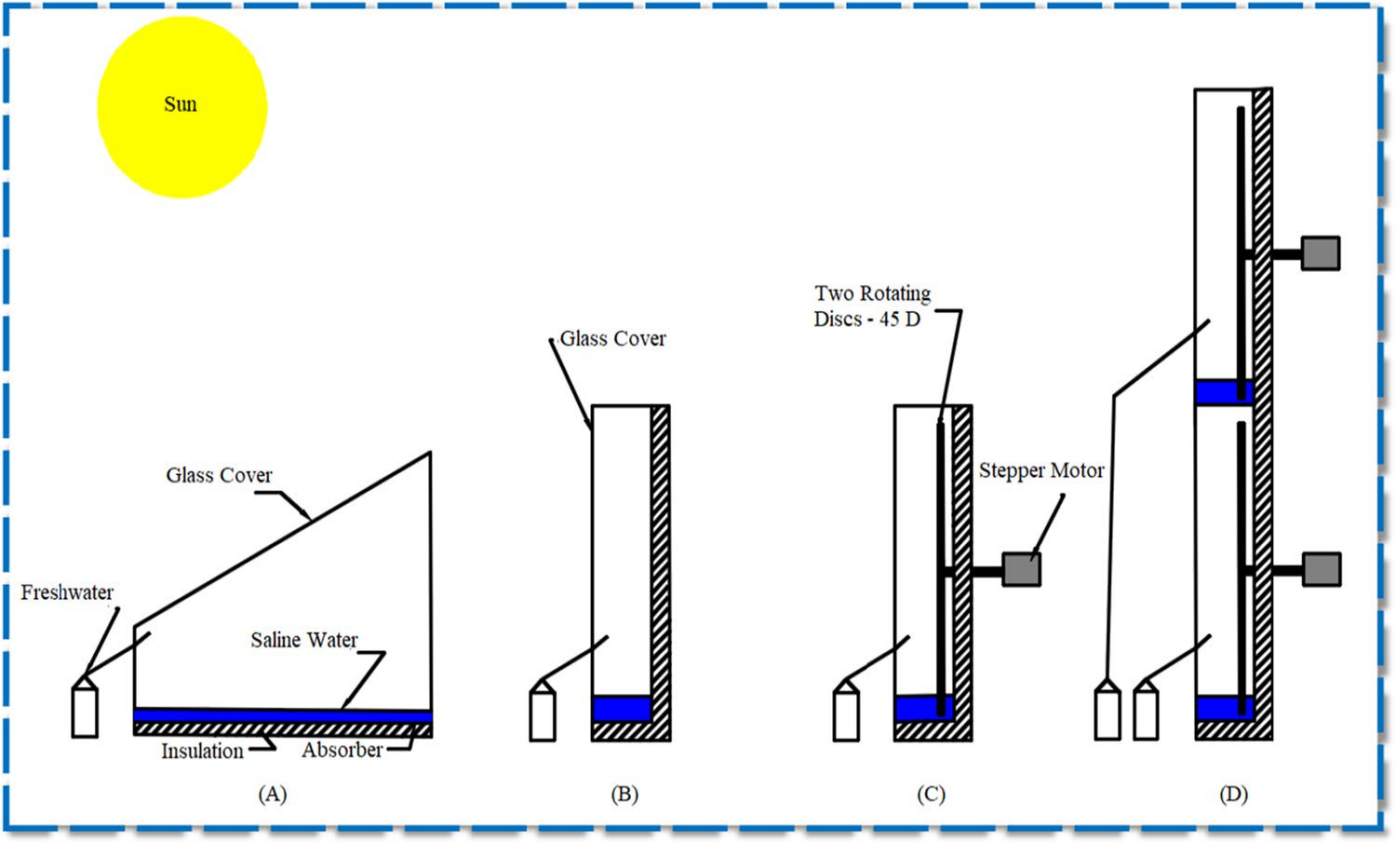
A 2D schematic drawing of the proposed distillers. **a** CTD. **b** CVD. **c** MSSVD. **d** MDSVD

CTD was planned for a 0.5 m^2^ projected area (50 cm wide and 100 cm long). The upper and lower sides of CTD were 0.43 m and 0.15 m, as shown in Fig. 2. It has also been built of 1.5 mm black-coated galvanized steel to make the best use of solar radiation absorbed. Afterward, the wooden frame and the outside part of the still reservoir were fitted with fiberglass (5 cm thickness). It acts as an isolating layer to prevent heat transfer from the standing to the outside. The outside dimensions of the wooden hollow box were 60 cm wide, 110 cm long, 33 cm high, and 15 cm low side. A 3-mm-thick glass layer was mounted on the CTD, and the inclination angle of glass cover was 31°. In addition, the concentrated droplets were collected in the distiller on a sloped channel and directed outwards of the basin to be accumulated on graduated flasks. The extra drain was handled manually using a pipe and valve fitted to the bottom of the distiller.

As seen in Fig. 2, the projected area of the CVD and MSSVD was 0.1 m^2^. They had a size of 10 cm wide with a length of 100 cm and a height of 50 cm. They were made of 2-mm-thick galvanized black paint steel to optimize solar radiation absorption. The L-sized and external scale wooden hollow box was 15 cm wide, 110 cm long, and 50 cm high. A 4-mm-thick glass sheet covered them.

Furthermore, the scholars also made specific designable modifications to the CVD. First, there are two movable discs installed on the back of the CVD as shown in Fig. 2. The moving discs had a circular shape of 45 cm in diameter (aluminum sheet 3 mm thick). Rotary discs were connected with a bearing bracket to the moving shafts. A small 6 W DC motor was required in order to turn the disc utilizing a set of pulley and belt mounting, 23 cm high from the bottom, 3 cm high from each side. A speed controller was used to adjust the speed of the rotating discs, which were coated in black to improve solar ray absorption. Second, MDSVD has been seen in Fig. 2 as double stages. It was 10 cm wide, 100 cm long, and 100 cm high. Third, the discs were manually operated by a controller. The control system is composed of a power supply, a 3-way switch, a Blue-LED, and a motor stepper, control, and synchronization circuit. Finally, the impact of varying water depth on the performance of solar distillers was examined at the optimum speed of 1.5 rpm. The water depth was changed from 5 to 14 cm (5, 8, 11, and 14 cm).

### Experimental tests

In Kafrelsheikh, Egypt (latitude = 31.1107° N and longitude = 30.9388° E), observational tests were conducted. In September 2020, the experiments were performed. All the distillers studied were mounted on the east–west axes to absorb as much of the radiation as possible from the sun. At the same time, parameters were calculated that influence the efficiency of the solar still, such as solar radiation, the temperature of the outside glass, water, ambient temperature, and airspeed. Furthermore, the water yield distilled hourly was noted. Equitable operational and environmental conditions were laid down for all stills.

The lower section of the disc was immersed in the reservoir in the morning. Thus, the discs started to rotate at the specified speed. Therefore, the higher section of the disc is the lowest section with rotation and conversely. As a result, the surfaces of the disc formed a thin layer of water. This film was quickly evaporated because warming does not take much time. The pure water was also collected and recorded daily. The experiments investigated the effects of various water depths (5, 8, 11, and 14 cm) on the solar still productivity at 1.5 rpm. Every depth was examined experimentally over the course of a single day.

### Experimental error analysis and measuring instruments

The devices utilized to measure the parameters impacting the performance of the solar distiller are datalogging solar power meter, K-type thermocouples, van-type anemometer, and graded bottles. The sun’s radiation was measured using the Datalogging solar power meter. Measuring temperatures at several distillation positions was done via K-type thermocouples. A van anemometer was also used to measure the speed of the air. Furthermore, the distillate was measured using a calibrated tiny flask. The specifications of the measuring instruments were indicated in Table 1.

**Table 1:** Measurement precision and experimental uncertainty errors

Instrument name	Units	Precision	Range		Error (%)
			Start from	Up to	
Datalogging solar power meter	W/m^2^	± 1	0	5000	1.5
K-type thermocouples	°C	± 0.1	0	100	1.3
Van-type anemometer	m/s	± 0.1	0.4	30	3
Graded flask	m L	± 1	0	2000	2

The approach given by Holman (2012) was used to evaluate the uncertainty in the experimental measurements. The following formula can be used to estimate the result errors:

1 $${W}_{R}= \sqrt{{\left(\frac{\partial R}{\partial {x}_{1}}{W}_{1}\right)}^{2}+{\left(\frac{\partial R}{\partial {x}_{2}}{W}_{2}\right)}^{2}+\dots +{\left(\frac{\partial R}{\partial {x}_{n}}{W}_{n}\right)}^{2}}$$

where W_R_ represented the resulting uncertainty and W_1_, W_2_, W_3_, …, W_n_ reflected the uncertainty in the independent variables. Table 1 showed the characteristics of the measurement equipment. In addition, the hourly output could be represented as a function of basin saltwater depth; *m* = *f* (h). Therefore, the following is the level of uncertainty for productivity:

Furthermore, the thermal efficiency uncertainty is as follows:

2 $${W}_{m}= \sqrt{{\left(\frac{\partial m}{\partial h}{W}_{h}\right)}^{2}}$$

3 $${W}_{{\eta }_{th}}= \sqrt{{\left(\frac{\partial {\eta }_{th}}{\partial m}{W}_{m}\right)}^{2}+{\left(\frac{\partial {\eta }_{th}}{\partial {I}_{R}}{W}_{{I}_{(t)}}\right)}^{2}}$$

Consequently, daily efficacy and productivity errors in stills are around ± 2.5% and ± 1.4%, respectively.

## Results and discussion

At different water depths, the effect of the rotating disc adjustment on the thermal efficiency of the vertical distillers was examined at the best speed (1.5 rpm). The current performance of distillers was assessed by conducting the behavior of various parameters, including solar radiation, temperature, and daily output of potable water. In addition, CTD and CVD efficiency was tested to compare with that of modified rotating disc solar stills.

### Performance of modified solar stills with rotating discs at different water depths

The research was concerned with highlighting the thermal efficiency (solar radiation, temperature, and productivity) of distillers at variable water depths (5, 8, 11, and 14 cm) at the best speed (1.5 rpm) to examine the influence of water depth varying on the output of stills. The indicated findings were at the best rotational speed (1.5 rpm) to find out the influence of disc adjustment and glass cover on distillers’ output and to prevent duplication. In addition, Table 2 tabulated the weather parameters (solar radiation, ambient temperature, and air speed) during the testing days.

**Table 2:** The measured factors of the solar distillers for the different testing days

Date	22/09/2020		rpm = 1.5			Water depth = 5 cm					
Time	Air speed, (m/s)	Ambient temperature, (°C)	Intensity of solar radiation (W/m^2^)					Accumulated yield, (mL/m^2^ day)			
	V	Ta	I top	I forward	I left	I right	I inclined	CTD	CVD	MSSVD	MDSVD
9:00	2.2	29.1	575.0	500.0	662.0	170.0	690.0	0	0	0	0
10:00	2.2	31.8	680.0	585.0	445.0	195.0	833.0	30	70	850	1300
11:00	0.8	33.1	770.0	630.0	332.0	215.0	893.0	90	210	1950	3100
12:00	3.6	34.2	792.0	705.0	222.0	260.0	914.0	260	460	3300	5350
13:00	2.5	35.6	740.0	566.0	215.0	459.0	840.0	480	760	4850	7850
14:00	2.7	34.8	595.0	450.0	186.0	590.0	662.0	800	1170	6200	10,100
15:00	3.0	33.9	480.0	377.0	186.0	675.0	530.0	1080	1530	7350	11,950
16:00	3.3	33.1	280.0	222.0	120.0	570.0	305.0	1320	1810	8350	13,500
Date	27/09/2020		rpm = 1.5			Water depth = 8 cm					
Time	Air speed, (m/s)	Ambient temperature (°C)	Intensity of solar radiation (W/m^2^)					Accumulated yield (mL/m^2^ day)			
	V	Ta	I top	I forward	I left	I right	I inclined	CTD	CVD	MSSVD	MDSVD
9:00	2.7	30.0	610.0	495.0	662.0	190.0	750.0	0	0	0	0
10:00	1.5	31.0	725.0	590.0	501.0	217.0	850.0	30	65	600	900
11:00	2.3	32.1	800.0	680.0	380.0	261.0	950.0	100	215	1700	2620
12:00	3.1	35.0	880.0	730.0	300.0	340.0	1025.0	275	485	3100	4820
13:00	0.9	34.0	810.0	630.0	261.0	526.0	920.0	531	895	4680	7320
14:00	4.1	32.5	700.0	510.0	225.0	640.0	790.0	876	1375	6330	9920
15:00	3.5	31.0	500.0	380.0	180.0	710.0	550.0	1186	1775	7810	12,210
16:00	3.1	30.3	307.0	240.0	135.0	622.0	320.0	1456	2065	8610	13,460
Date	28/09/2020		rpm = 1.5			Water depth = 11 cm					
Time	Air speed, (m/s)	Ambient temperature (°C)	Intensity of solar radiation (W/m^2^)					Accumulated yield (mL/m^2^ day)			
	V	Ta	I top	I forward	I left	I right	I inclined	CTD	CVD	MSSVD	MDSVD
9:00	2.4	30.0	620.0	525.0	735.0	194.0	790.0	0	0	0	0
10:00	1.4	31.3	710.0	570.0	545.0	221.0	875.0	28	58	500	700
11:00	2.7	33.0	814.0	660.0	385.0	265.0	952.0	104	198	1500	2200
12:00	3.0	36.4	900.0	770.0	310.0	344.0	1010.0	282	478	2850	4290
13:00	1.4	35.5	792.0	633.0	270.0	530.0	885.0	542	893	4380	6770
14:00	2.8	34.0	670.0	510.0	228.0	644.0	730.0	882	1393	5980	9310
15:00	3.3	33.2	466.0	330.0	188.0	714.0	572.0	1197	1813	7410	11,560
16:00	3.3	31.8	295.0	245.0	130.0	626.0	325.0	1477	2123	8390	13,110
Date	29/09/2020		rpm = 1.5			Water depth = 14 cm					
Time	Air speed, (m/s)	Ambient temperature (°C)	Intensity of solar radiation (W/m^2^)					Accumulated yield (mL/m^2^ day)			
	V	Ta	I top	I forward	I left	I right	I inclined	CTD	CVD	MSSVD	MDSVD
9:00	2.2	31.0	635.0	540.0	655.0	210.0	745.0	0	0	0	0
10:00	0.7	31.9	765.0	630.0	505.0	240.0	875.0	26	50	300	460
11:00	1.3	33.4	890.0	700.0	377.0	261.0	970.0	82	175	1070	1580
12:00	2.5	36.5	950.0	730.0	300.0	340.0	1020.0	238	415	2320	3510
13:00	1.8	35.3	820.0	630.0	261.0	526.0	905.0	528	795	3970	6010
14:00	3.9	33.1	720.0	525.0	245.0	700.0	800.0	908	1305	5670	8730
15:00	3.5	32.8	500.0	380.0	180.0	740.0	554.0	1268	1775	7090	10,960
16:00	3.3	31.7	305.0	238.0	135.0	660.0	338.0	1563	2115	8290	12,760

**Fig. 3:**
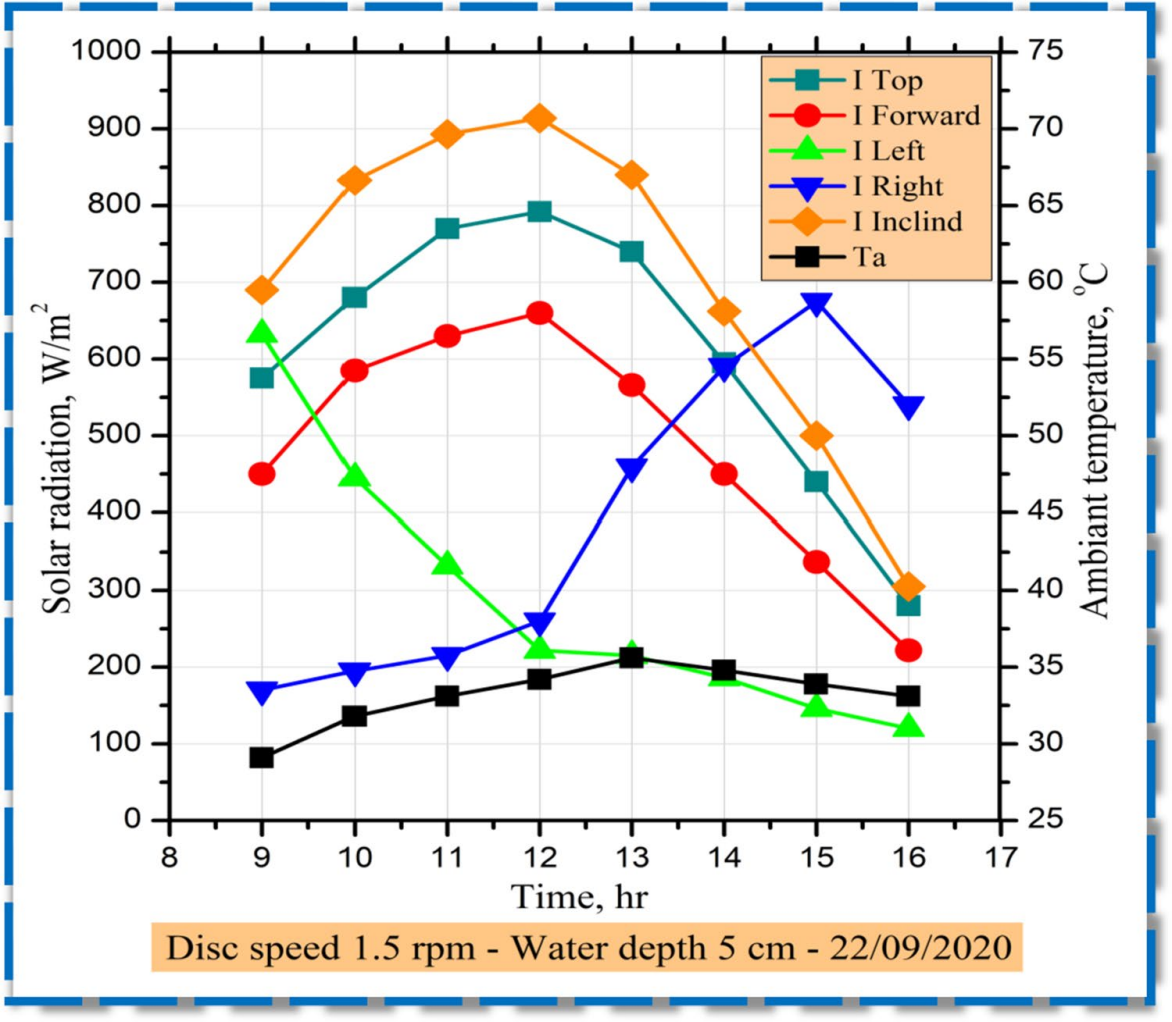
Recoded values of hourly radiation and ambient temperature variations on solar stills

Radiation from the sun rose steadily between sunrise and midday with the highest value (910 w/m^2^ at midday). Therefore, its amount steadily declined till it was minimum as shown in Fig. 3. At sunset, the intensity of solar radiation was variable from side to side, attributable to the glass cover configuration for absorbing extra rays. Solar radiation was consequently estimated on both the front, top, left, right, and tilted faces.

**Fig. 4:**
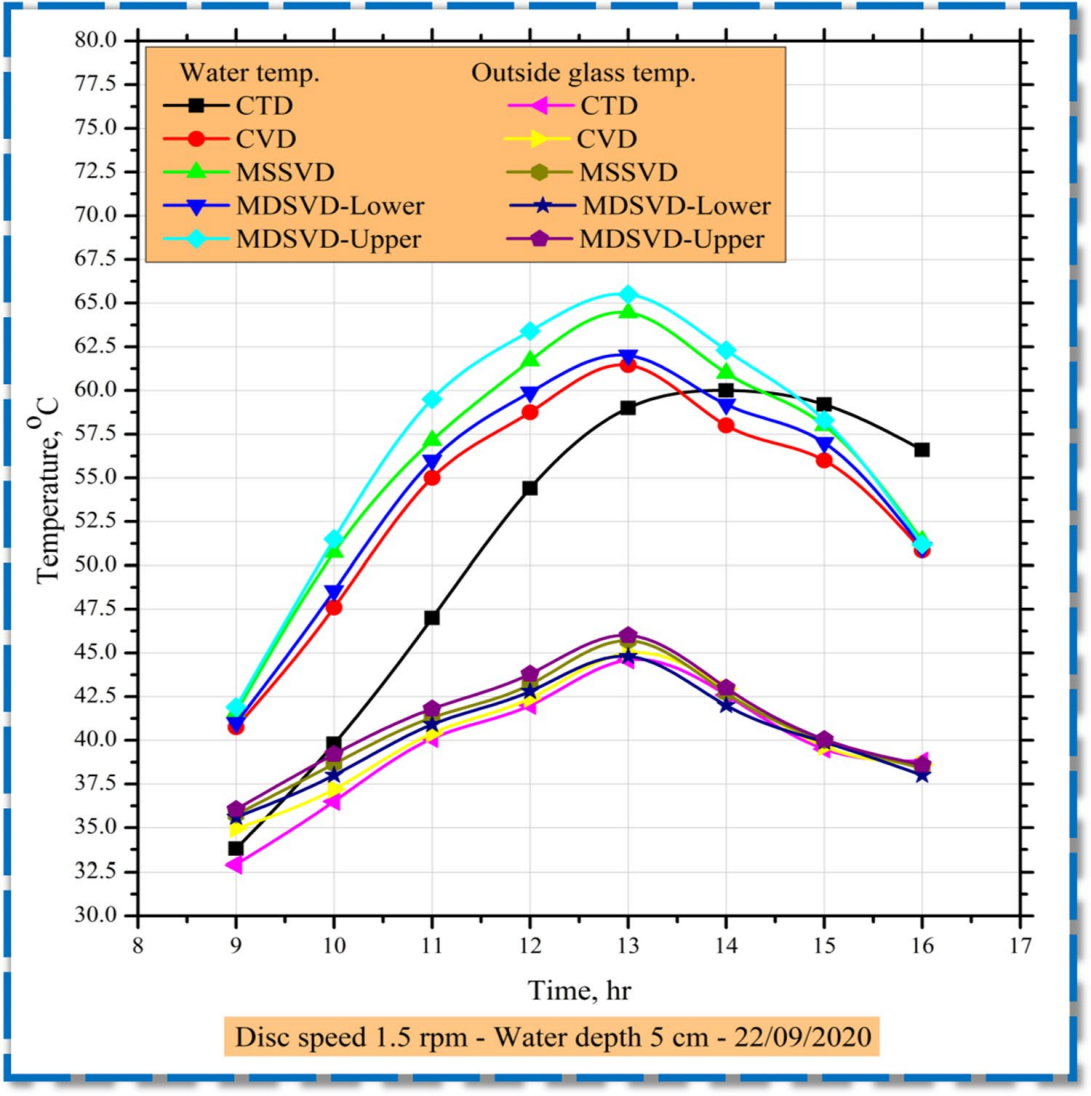
Hourly variations of water and outside glass temperature on distillers

The water and glass cover temperatures in the still basin had the same solar radiation differences as seen in Fig. 4. In the vertical distillers, the water temperature is about 0–10 °C higher than the CTD temperature. Due to its exposure to the water directly within the vertical distiller, owing to tracking by the Sun, the transmission of solar radiation also leads to water heating, while in the CTD, the water basin is only warmed by the solar rays directly transmitted. In vertical distillers, the temperature of saltwater is larger than the temperature of CTD. In contrast, for solar stills at 16:00, water temperatures were nearly the same. By comparison, while the vertical distillers’ water temperature is generally higher than that of CTD, the vertical distiller’s glass temperature is higher than the CTD's. It is because the discs have already generated high evaporation rates. In general, glass temperature is around 0–3 °C above CTD in the enhanced distillers.

**Fig. 5:**
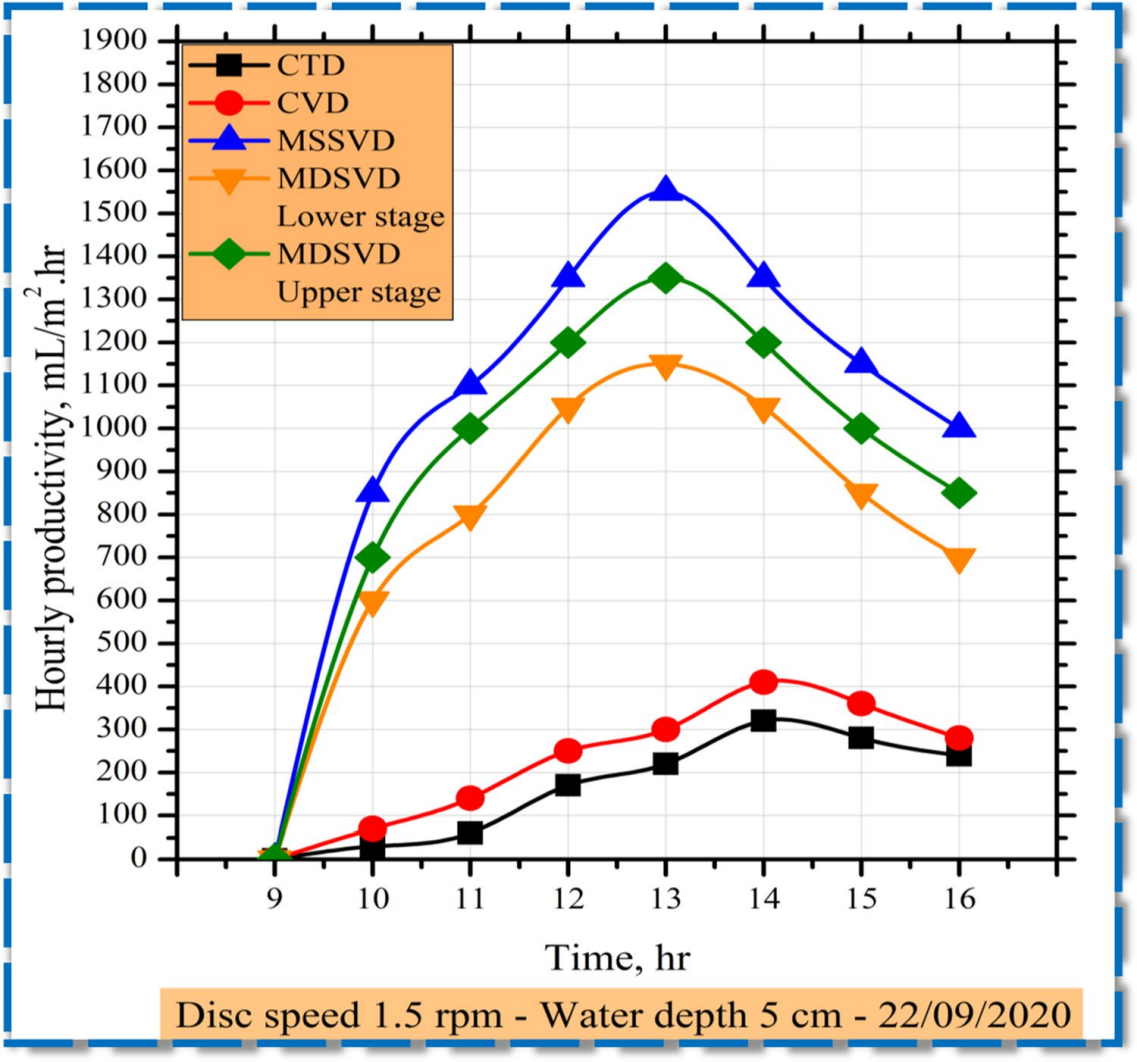
The hourly productivity variations for solar stills at the rotational speed of 1.5 rpm

The hourly difference in yield for solar stills is seen in Fig. 5 at 1.5 rpm. The distiller output is low in the morning because there has been no warming of discs and saltwater and there is a need to warm up the cool water. After that, the distillation starts with solar radiation to record highest values at 13:00 [1550 and 2500 mL/m^2^ h] for MSSVD and MDSVD, and [320 and 410 mL/m^2^ h] for CTD and CVD at 14:00.

The distillation has been significantly enhanced in the scenario of discs stills. Subsequently, as a result of solar intensity behavior, the amount of distilled water gradually decreases. Quantitatively, the distiller’s hourly productivity is higher than the CTD’s as seen in Fig. 5. This rise has four main causative factors as follows:

1. On the disc surface, the film layer of water is thin; therefore, the evaporation rate is significantly higher than CTD.
2. The wide surface area for vertical distillers exposed to sun energy strengthens the mechanism of evaporation within the modified distillers. For comparison, the evaporative zone of the CTD is 0.5 m^2^ while the CVD is 0.1 m^2^. The area of the MSSVD is 0.74 m^2^ (0.1 m^2^ and 0.16 m^2^ on every side of the disc), and an area of MDSVD of 1.48 m^2^ (0.74 m^2^ for each stage). In contrast, the disc surface areas exposed to solar radiation are nearly 0.32 m^2^, relative to the CTD region of just 0.5 m^2^. As a result, owing to the large evaporation area, the rate of evaporation in the MSSVD and the MDSVD is greater than that in the CTD.
3. The eddies that improve the evaporation mechanism has occurred within the modified stills connected with rotating discs. The disc drives movement over the water and the air in the basin. This means the extraction of the water vapor produced by the saltwater surface that would be collected within the glass shell.
4. Sun tracking vertical stills have collected a significant amount of solar rays all day. The surface area of exposure of the modified distillers is higher than the conventional. For illustration, the CTD’s area of exposure is 0.5 m^2^, whereas MSSVD and MDSVD are respectively 0.74 m^2^ and 1.48 m^2^.

The speed of 1.5 rpm as indicated above is ideal for continually preparing and evaporating the thin film of the water layer on the disc surface. This eliminates dry spots from forming on the disc surface. Therefore, as observed in Fig. 5, the disc distiller provided more drinking water than CTD.

**Fig. 6:**
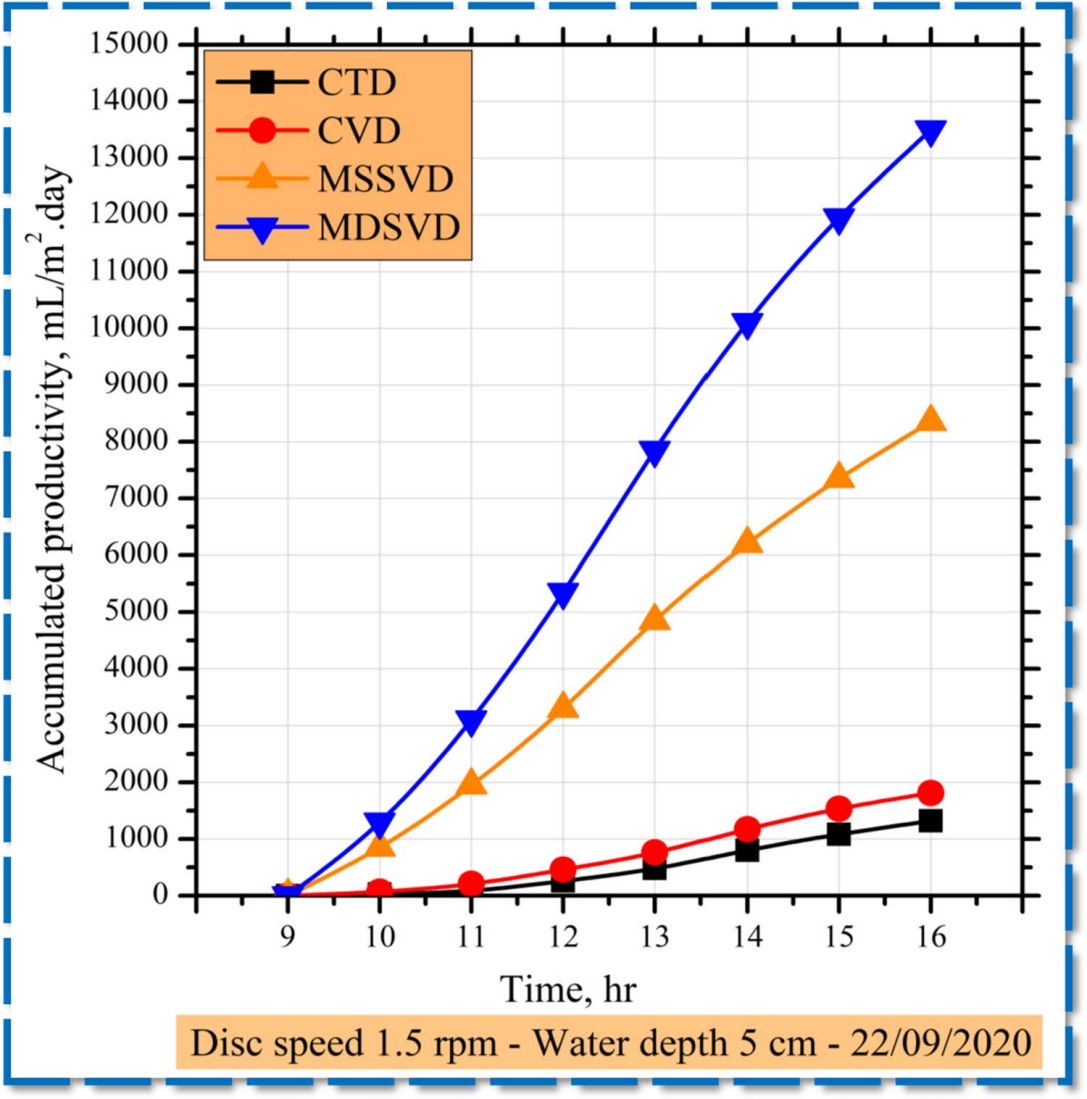
The hourly accumulated productivity of CTD, CVD, MSSVD, and MDSVD at 1.5 rpm

Productivity accumulated at 1.5 rpm for the CTD, CVD, MSSVD, and MDSVD as shown in Fig. 6. The disc still, as shown in the graph, provides more drinking water than CTD. The daily freshwater accumulated production is estimated for CTD of about 2300 mL/m^2^ day compared to 10,350 mL/m^2^ day for MSSVD with a rise of 350% and 16,500 mL/m^2^ day for MDSVD with a rise of 617.4%. The explanation is that the thin saltwater layer reduces the thermal capacity of the mass of water.

Furthermore, the rotating discs increase the evaporative surface area and thermal transfer parameters. Consequently, the usage of the disc improves the intensity of the transmitted solar energy correlated with the conventional still and thereby raises the evaporation of the saline water. The heat transfer rate in the disc distiller between the disc and the thin water film is extra than that between the water and the basin plate of the traditional still, therefore the productivity is enhanced.

### Daily productivity enhancement of stills at different water depths at the best disc speed of 1.5 rpm

The daily rise in solar still productivity is dependent on various operating parameters as follows:

1. The speed of rotating discs: the scholars showed in previous work (Essa et al. 2021) that the best disc speed was 1.5 rpm after investigating numerous values (starting from 0.125 to 2.5 rpm). The results revealed that because the quicker revolution of the discs provided little time for a completely evaporative water layer, the distillate yield was limited to a slower speed relative to 1.5 rpm. Furthermore, the discs could not absorb enough water and were always dry at low speeds, even 0.125 rpm.
2. Solar radiation: due to the four sides glass cover, the vertical stills captured more sun rays compared to CTD, which extremely enhanced the evaporation rate; hence, the productivity was increased.
3. Glass cover temperature: because of the large area of glass exposed to air, the difference in water–glass temperatures increases and the condensation area was maximized. Thus, the condensation rate was improved and led to productivity rise.
4. Saltwater depth: incorporating aluminum discs into vertical solar still increased the evaporation rate via increasing evaporation area and lessening the water layer. In addition, these discs broke down the water surface tension which facilitates the evaporation mechanism.

**Fig. 7:**
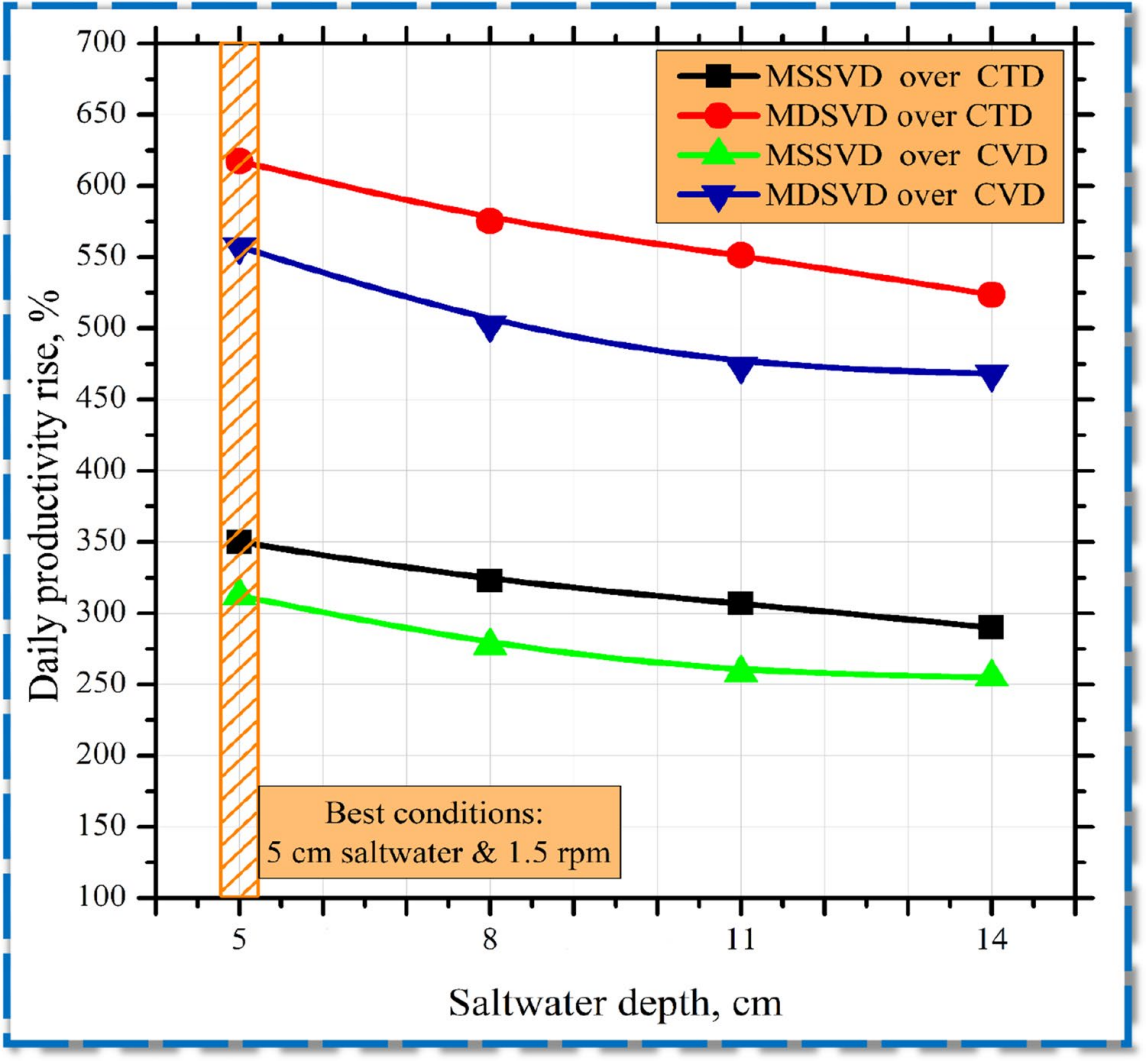
Daily yield rise of stills under numerous water depths at 1.5 rpm

As mentioned above, the authors selected the best operating speed to show the impact of varying water depths at the output of four stills as illustrated in Fig. 7. Although the wet space of the rotary disc increased, the distillate tends to decrease. That is because the basin had a more saltwater quantity that want more solar radiation to be warmed.

### Daily thermal efficacy

The daily distiller efficiency, $\upeta$_**d**_, is computed by multiplying the daily yield $(\sum \dot{\mathrm{m})}$ by the latent heat of vaporization ${\mathrm{h}}_{\mathrm{fg}}$. Later, the outcome is divided by the average daily solar intensity, I (t), across the projected area A of the still plus the total motor energy (Essa et al. 2020):

4 $$\upeta \mathbf{d}=\frac{\sum \dot{\mathrm{m}} \times {\mathrm{h}}_{\mathrm{fg}}}{\mathrm{\Sigma A }\times \mathrm{ I}\left(\mathrm{t}\right)+\mathrm{ Motor\;energy}}$$

**Fig. 8:**
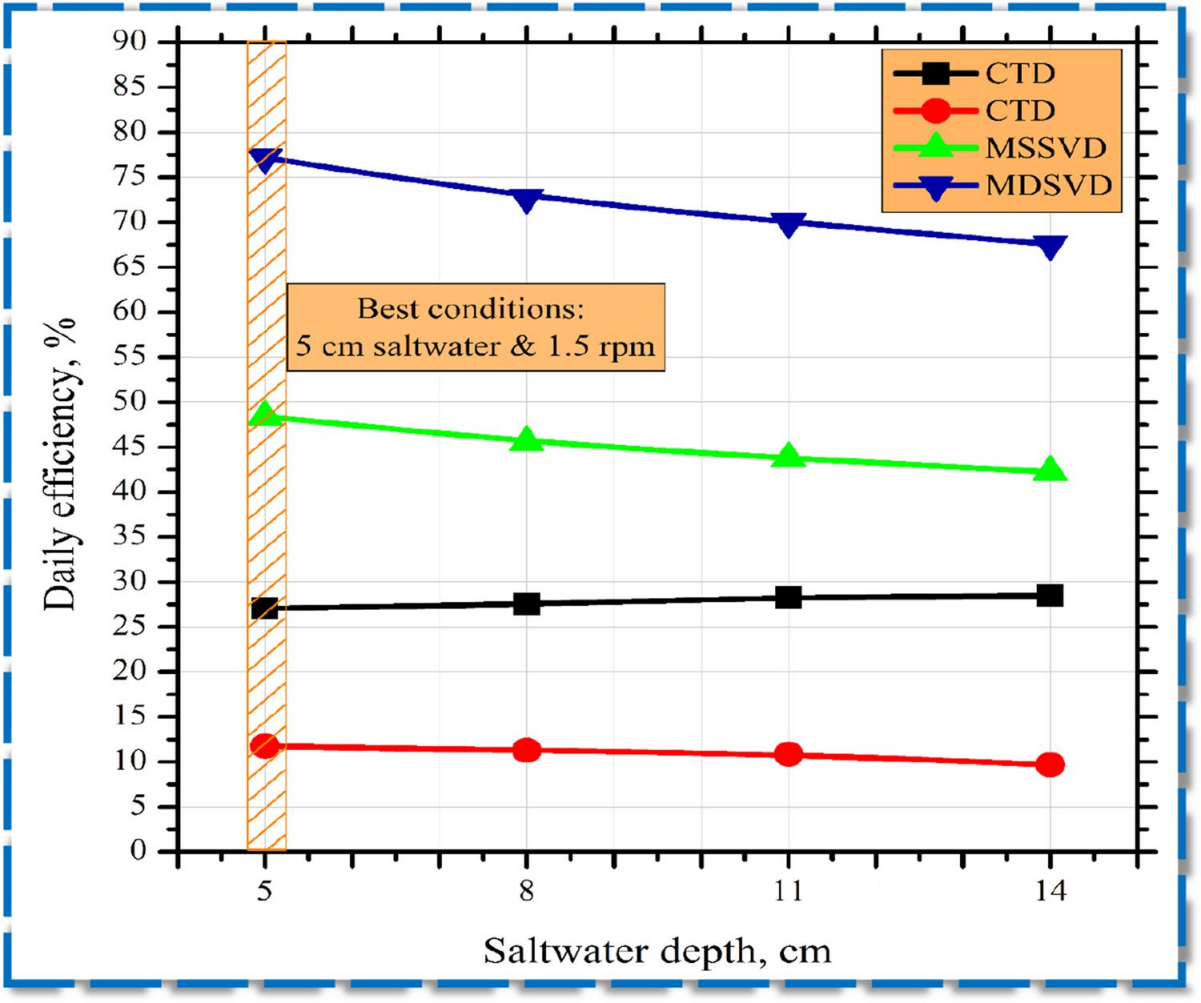
Daily efficacy of distillers at numerous saltwater depths

In the several cases analyzed, the daily efficacy of the investigated stills is demonstrated in Fig. 8. The thermal efficacy curves are shown to have the same pattern as in Fig. 7. For MSSVD and MDSVD, the superior thermal efficacy was 48.4% and 77.2% at 1.5 rpm and 5 cm saltwater depth. In contrast, the efficiency of CTD and CVD were about 27.1% and 11.7% respectively.

### Relation of the present work to previous studies

Earlier studies are compared to the current study’s findings to determine how much growth can be achieved with the modified vertical distiller, as demonstrated in Table 3.

**Table 3:** A comparison of the results of the current and previous studies

Classification	Authors	Improvement	Efficiency
Rotating disc distiller	Essa et al. (2020)	124%	54.5%
Drum distiller	Abdullah et al. (2021a)	296%	79%
	Abdullah et al. (2019b)	350%	85.5%
Solar distiller with moving wick	Abdullah et al. (2021b)	300%	82%
	Abdullah et al. (2019a)	315%	84%
	Haddad et al. (2017)	14.72% and 51.1% in summer and winter, respectively	65%
Solar distiller with water fan driven by a wind turbine	Omara et al. (2017)	17%	39.8%
	Kabeel et al. (2012)	25%	38%
Solar distiller with a rotating shaft	Kumar et al. (2016)	39.49%	30.57%
Solar distiller with vibratory harmonic effect	Eldalil (2009a, b; 2010)	132%	60%
Vertical solar still	Diab et al. (2022)	660.45%	84.05%
Current study	Effect of basin water depth on the performance of vertical discs’ solar still – Experimental investigation	617.4%	77.2%

### Cost evaluation of purified water

The cost calculation for the studied distillers focuses mostly on the distiller category as well as its components (Table 4).

**Table 4:** Solar stills manufactured expense for 1 m^2^

Unit	CTD ($)	CVD ($)	MSSVD ($)	MDSVD ($)
Iron sheet	30	90	90	165
Aluminum disc	–	–	20	40
Glass sheet	10	40	40	70
Ducts and support legs	25	35	35	45
Paint	10	15	20	30
Insulation (Fiberglass)	7	20	20	35
Production	20	20	30	50
DC-Motor and connections	–	–	45	80
Total fixed cost (P)	102	220	300	515

The analytical formulas (Abdullah et al. 2020) have been demonstrated to calculate the cost of pure water extracted by CTD, CVD, MSSVD, and MDSVD as indicated in Table 5.

**Table 5:** Economic study equations

Parameters	Equations	Declarations
The capital recovery factor	$$\mathrm{CRF }= \frac{{\mathrm{i}(1+\mathrm{i})}^{\mathrm{ n}} }{{(1+\mathrm{i}) }^{\mathrm{n}}-1}$$	▪n is the lifetime (years) ▪ “i” is the interest rate ▪P is the capital cost of solar still ($) ▪M is the average yearly distillate production
The fixed annual cost	$$\mathrm{FAC }=\mathrm{ P }(\mathrm{CRF})$$	
The sinking fund factor	$$\mathrm{SFF }= \frac{\mathrm{i }}{{(1+\mathrm{i}) }^{\mathrm{n}}-1}$$	
The salvage value	$$\mathrm{S }= 0.2\mathrm{P}$$	
The annual salvage value	$$\mathrm{ASV }=\mathrm{ S}(\mathrm{SFF})$$	
The annual maintenance and operating costs	$$\mathrm{AMC }= 0.15(\mathrm{FAC})$$	
The total annual cost	$$\mathrm{TAC }=\mathrm{ FAC }+\mathrm{ AMC}-\mathrm{ ASV}$$	
The cost of freshwater $/L	$$\mathrm{CPL }=\mathrm{ TAC}/\mathrm{M}$$	

For the cost analysis, the following are taken into account: the number of working days a year is 340 days, the rate of interest is 15%, and the still lifetime is 10 years. For CTD, CVD, MSSVD, and MDSVD, the average daily output is roughly 2.663, 2.983, 10.81, and 17.26 L/m^2^ day respectively. Economic analysis indicates that drinking water costs provided by CTD, CVD, MSSVD, and MDSVD are respectively 0.025, 0.0477, 0.0180, and 0.0193 $/L.

## Conclusion

Experimentally and compared to the traditional distiller, the performance of vertical solar still incorporated with rotational discs is studied. This research investigated the key parameters, which consisted of best disc speed and saltwater depth variations. The findings discussed above, which are primarily based on, are clarified that:

1. The installation of rotating discs in the distiller significantly improved the production of purified water and the efficiency of thermal energy leading to rising evaporative surfaces. In addition, attributable to sun tracking, the area of exposure was improved.
2. The highest yield values were obtained for the modified stills at 1.5 rpm and 5 cm water depth, whereby MSSVD and MDSVD purified water distillate were respectively enlarged by 350% and by 617.4% over CTD. And it was respectively increased by 312.4% and 557.4% over CVD.
3. The best efficacy for MSSVD and MDSVD was 48.4% and 77.2% at 1.5 rpm and 5 cm water depth, respectively. While CTD and CVD had an efficacy of around 27.1% and 11.7%, respectively.
4. The costs of clean water gained from CTD, CVD, MSSVD, and MDSVD are calculated to be 0.025, 0.0477, 0.0180, and 0.0193 $/L, respectively.

### Scope for future work

There are several causes why solar distillers with moving elements should indeed be investigated more thoroughly:

- Different rotating disc configurations such as corrugated and finned discs are being studied to enhance absorption and evaporation rates.
- Examining the impact of pulsed water sprayer on still performance while the saltwater depth is minimum.
- PCM and nanomaterials (such as CuO and AL_2_O_3_) can be used to store energy and enhance the performance of the distiller.

## Data Availability

Not applicable.

## Abbreviations

CTD: Conventional tilted distiller
CVD: Conventional vertical distiller
MDSVD: Modified double-stage vertical distiller
MSSVD: Modified single-stage vertical distiller

## Nomenclature

A: Projected area of still, (m^2^)
AMC: The annual maintenance and operating costs, ($)
ASV: The annual salvage value, ($)
CPL: The freshwater cost, ($/L)
CRF: The capital recovery factor
F: The distiller capital cost, ($)
FAC: The fixed annual cost, ($)
hfg: Latent heat of vaporization, (KJ/kg)
i: The interest rate, (%)
I(t): Solar intensity, (W/m^2^)
$$\dot{\mathrm{m}}$$: Hourly distillate yield, (mL/m^2^.hr)
M: The average freshwater yield in year, (L/m^2^ year)
n: The still lifetime, (years)
S: The salvage value, ($)
SFF: The sinking fund factor
TAC: The total annual cost, ($)
η: Efficiency
